# The Benefits of Being Proactive While Working Remotely: Leveraging Self-Leadership and Job Crafting to Achieve Higher Work Engagement and Task Significance

**DOI:** 10.3389/fpsyg.2022.833776

**Published:** 2022-04-25

**Authors:** Arianna Costantini, Jared Weintraub

**Affiliations:** ^1^Department of Psychology and Cognitive Science, University of Trento, Rovereto, Italy; ^2^The Flow Group, LLC, New York, NY, United States

**Keywords:** remote work, self-leadership, job crafting, task significance, COVID-19, work engagement

## Abstract

Given the growing number of remote and hybrid working arrangements, this research investigates the process and outcomes of proactivity during remote work. We approach proactivity during remote working as a resource-building process and integrate self-leadership and job crafting literature. We propose that employees’ self-leadership allows them to regulate their resources optimally, enabling resource availability that can be used to arrange remote working demands and resources proactively. We collected three-wave data from remote workers (*n* = 329 observations) and tested our hypotheses using multilevel analyses. Results differed by level of analysis. Specifically, at the between level, comparing behaviors between participants, social expansion mediated the relationship between self-goal setting and task significance. In contrast, at the within level (analyzing differences in behavior within the same person), social expansion mediated the relationship between self-goal setting and work engagement. Overall, these findings suggest that self-leadership allows higher availability of resources enabling the proactive initiation of social interactions, which, at the within level enhance work engagement, and at the between level improve task significance during remote work. We discuss these findings considering the implications for interventions to foster more positive remote-work experiences.

## Introduction

The outbreak of the COVID-19 pandemic led millions of people across the world into remote work, with remote and hybrid working arrangements becoming the “new normal” almost overnight (Wang et al., 2020; Kniffin et al., 2021; Becker et al., 2022). This situation poses new challenges to understanding the processes and outcomes of remote work since it is no longer based on individual arrangements and specific requests but represents an entirely new context of work (Wang et al., 2020). To address these challenges and further knowledge on the new context of work, researchers started investigating how “virtual” work characteristics shape work, with remote work understood as a setting that profoundly re-shapes work characteristics and experiences (Bailey and Kurland, 2002; Wang et al., 2020).

Literature provides initial evidence for the importance of self-discipline as a means of dealing with the challenges of remote work and mitigating the demands which undermine employee wellbeing (Wang et al., 2020). Yet, the understanding of how people manage themselves when working remotely has been largely omitted in previous studies and is particularly limited when considering the new ways of working that took shape after the outbreak of COVID-19 (Wang et al., 2020; Becker et al., 2022). This lack of knowledge on how proactivity unfolds during remote work after COVID-19 is particularly relevant. Working remotely represents a “weak” situation, where employees have high levels of autonomy, the goals (nor the means to achieve them) are not clearly specified, and the attainment of these goals is often unlinked to predefined rewards (Mischel and Shoda, 1995). Moreover, given that this new context of work seems characterized by less frequent interactions between leaders and employees (Gibbs et al., 2021) and that close monitoring during remote work is shown to have adverse effects on employees’ wellbeing (Wang et al., 2020), it is crucial to gain a better understanding of the role of self-leadership for proactivity during remote work after COVID-19.

In this study, we use a quantitative diary approach and follow remote workers weekly to investigate how their strategies to lead and manage themselves toward performance during remote working enable higher resources to craft their work and experience higher work engagement and task significance. We build on self-regulatory and proactivity research to integrate self-leadership (Manz, 1986; Houghton and Neck, 2002) and job crafting literature (Wrzesniewski and Dutton, 2001), and propose that employees’ self-leadership allows them to optimally regulate their resources. We further propose that this process enables resource availability that can be used to proactively arrange remote-work characteristics (i.e., job crafting), which leads to positive remote-work experiences.

We aim to contribute to the literature as follows: First, we integrate self-leadership and job crafting research, and place it in the context of remote working during the pandemic. We argue that when remote workers use self-leadership strategies more often, they are better equipped to proactively craft their virtual work environment, leading to higher wellbeing. In doing so, this research offers a better understanding of the role of self-leadership in improving remote workers’ ability to deal with and positively alter their work environment proactively. Unpacking such processes of self-leadership and proactivity, and their link with work-related wellbeing during remote working is timely and relevant. Remote-work arrangements are likely to be used much more in the future, with positive net effects depending on whether they are implemented well (Gibbs et al., 2021).

Second, we contribute to job crafting research by investigating the mediating role of job crafting as a factor linking self-leadership, work engagement, and task significance in the context of remote working. Although job crafting research has to date acknowledged that job crafting arises from the interplay between a person and his/her work context (Parker et al., 2010), previous studies accounted for the role of (external) leadership (i.e., Lichtenthaler and Fischbach, 2018; Thun and Bakker, 2018), but only partially for self-influencing strategies that help people to take charge of their own motivation and performance (Neck and Houghton, 2006). Shedding light on the link between self-leadership and job crafting during remote working is important for the development of work proactivity research, for HRM practices to discover which self-regulation strategies enable individuals to better deal with their “virtual” work environment and to gain a better understanding of the underlying mechanisms that make them effective. While studies in traditional working contexts have established the link between job crafting and work engagement (*cf.* Zhang and Parker, 2019; Costantini et al., 2021), to the best of our knowledge, the effects of job crafting on task significance during remote working remain unexplored. Task significance refers to the degree to which employees perceive their job has a substantial impact on the lives or work of other people, whether in the immediate organization or in the external environment (Hackman and Oldham, 1975). Understanding the link between job crafting and task significance during remote working is relevant to shed light on whether and how job crafting has the potential to alter employees’ perceptions and (re)interpretation of the significance of their work (Wrzesniewski et al., 2013) when working from home. This is important to provide avenues for remote-work design and HRM policies and practices aimed at sustaining one’s sense of purpose, an aspect that, research shows, can be negatively impacted when remote working is suddenly introduced amid crisis (Ouwerkerk and Bartels, 2020).

**Figure 1:**
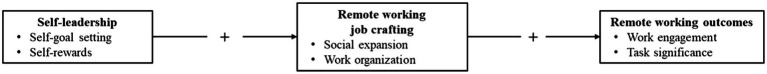
Model of remote working proactivity processes and outcomes.

Third, by using a diary method, we examine the dynamics of self-regulatory processes during remote working between individuals, as well as how an individuals’ own experience changes over time. Namely, we shed light on how job crafting leads to work engagement and task significance based on general differences between people and weekly changes in individuals’ experiences. In doing so, we complement the study of between-person differences with a within-person approach with the aim of enriching the literature on proactive work design for positive remote working experiences. Hence, this study advances knowledge on whether the proactive and self-regulatory processes during remote work are consistent—homologous—across different levels of analysis (*cf.* Gabriel et al., 2019), improving the understanding and theoretical development of job crafting and self-leadership literature. Recent cross-sectional research conducted during the pandemic suggested that self-discipline may be an important factor for remote workers to utilize the social resources from work to reduce loneliness (Wang et al., 2020). In the present research, we advance such literature by presenting three-wave longitudinal data and shed light on how weekly variations from employees’ baseline use of self-leadership strategies during remote working have implications for their involvement in job crafting—including the proactive initiation of social interactions—and its resulting outcomes. Such an analysis is relevant to inform theory and policy development by showing how self-leadership behaviors relate to job crafting variations during remote working, considering differences between people, while also examining weekly variations in individual experiences. Figure 1 shows the overarching model of the present study.

## Theoretical Background

### Self-Leadership

Self-leadership is a process through which individuals exert self-influence over their thoughts, feelings, and behaviors at work (Harari et al., 2021). Drawing on insights from classical self-regulation and self-control theories (Bandura, 1986; Carver and Scheier, 1998), self-leadership theory proposes that self-influence strategies serve to establish intrinsic motivation, resulting in enhanced individual performance (Manz, 1986). Specifically, the self-leadership perspective emphasizes that individuals self-direct themselves not only to achieve externally defined goals and standards, but also to self-influence and establish intrinsic motivation leading to desired performance results (Manz, 1986). Through self-leadership, people achieve the self-direction and self-motivation necessary to perform (Neck and Houghton, 2006, p. 271). Hence, self-leadership strategies allow employees to engage in activities they *want to*—rather than they only feel they *should*—perform (Manz, 1986).

Existing literature recognizes three self-leadership strategies that can be used to achieve self-direction and motivation (Houghton and Neck, 2002; Harari et al., 2021): behavior-focused strategies, constructive thought pattern strategies, and natural reward strategies. Specifically, *behavior-focused* strategies enhance self-awareness for the management of one’s behaviors, *constructive thought pattern* strategies center on forming habitual constructive thoughts emphasizing positive outcomes, and *natural reward* strategies emphasize the enjoyable aspects of a given task or activity (Houghton and Neck, 2002; Harari et al., 2021). Meta-analytic evidence shows that these various self-leadership strategies contribute differently toward particular outcome variables, with behavioral strategies contributing more toward regulating behavioral outcomes (Harari et al., 2021). Since in this study we are interested in understanding how self-leadership contributes to employees’ proactive behaviors during remote working, we focus on self-leadership behavioral strategies. In the context of COVID-19, characterized by a widespread and abrupt change to remote work (Becker et al., 2022), we expect individuals who could effectively set goals for themselves and reinforce their own positive, desirable behaviors during remote working to be better equipped to initiate the proactive redesign of their remote—or virtual—work characteristics.

### Job Crafting

Self-leadership literature recognizes that individuals may re-frame certain aspects of the performance process to establish enhanced motivational potential for work performance (Manz, 1986, p. 594). This, in turn, may serve to prompt proactive job redesign to improve the fit between the individual and the job when employees transform their work motivation into desired behaviors (Zeijen et al., 2018). In other words, we propose that while self-leadership defines the self-influencing process prompting individuals to redefine certain aspects of the performance process to *build intrinsic motivation*, once those self-leadership strategies are activated, individuals may follow up by engaging in job redesign efforts that focus on *redefining the job characteristics* to make their work better fit their own needs and preferences.

We shed light on these dynamics and dig into such performance and motivational enhancing processes by investigating how self-leadership prompts proactive work redesign in terms of job crafting behaviors during remote working. Job crafting describes the proactive, self-initiated changes in job boundaries aimed at improving one’s job and finding more meaning in it (Wrzesniewski and Dutton, 2001; Bruning and Campion, 2018). Research shows that job crafting can take several forms: employees may alter the number of tasks they have or the content of these tasks, they may change the amount and intensity of the relationships they have at work, or they may re-frame their thoughts about the aspects that give meaning to their job (Wrzesniewski and Dutton, 2001; Tims and Bakker, 2010). Importantly, these strategies do not refer to the redesign of the job as a whole, but to changing certain aspects or making small alterations that can impact the achievement of work goals (Tims and Bakker, 2010). Literature on job crafting shows that many of these strategies focus on active changes to one’s job to achieve future-oriented goals—also referred to as approach-oriented job crafting (Bruning and Campion, 2018)—which result in optimized work environments leading to higher work engagement and performance outcomes (Bakker and Oerlemans, 2019; Zhang and Parker, 2019; Costantini et al., 2021).

In this study, we focus on two job crafting strategies that reflect active, effortful, problem-focused, and improvement-based goals. These approaches are referred to as work organization and social expansion strategies (Bruning and Campion, 2018). *Work organization* involves the active design of systems and strategies to organize the tangible elements of work and can include managing behavior or physical surroundings to increase structural job resources (Tims et al., 2012; Bruning and Campion, 2018). Examples of work organization are making sure of having one’s tools laid out and ready to be used for work, organizing procedures, adding or dropping tasks, reviewing, and preparing the upcoming bundle of tasks (Wrzesniewski et al., 2013; Bruning and Campion, 2018). Differently, *social expansion* occurs within the social domain of work and involves changing the scope, number, and nature of social relationships within one’s work. Behaviors in this domain involve systematic feedback-seeking or changing how one interacts with others, also changing the boundaries around social activities. For example, in order to get the work done, employees may find ways to relate to their co-workers by getting to know them better, spending more time with the preferred ones, or seek support from people in the work environment (Wrzesniewski et al., 2013; Bruning and Campion, 2018; Breevaart and Tims, 2019). In the context of the pandemic, these relational proactive behaviors are particularly relevant because they increase feelings of social connectedness and provide additional opportunities to stay socially connected, despite spatial dispersion and isolation (Kniffin et al., 2021; Rudolph et al., 2021).

### Self-Leadership and Job Crafting

As a proactive behavior, job crafting is part of a goal-driven process involving setting a proactive goal and striving to achieve it (Parker et al., 2010). Specifically, proactive goal generation consists of envisioning and planning a goal under one’s own volition meaning that proactive goal generation is self-initiated and signals psychological ownership of change (Wagner et al., 2003; Parker et al., 2010). Previous research shows that individuals with long-term goals and a focus on growth are more likely to engage in job crafting later (Kooij et al., 2017a) and that self-goal setting positively mediates the motivating power of work engagement on job crafting (Zeijen et al., 2018). Accordingly, we expect employees scoring high on self-goal setting to be stimulated to craft their work proactively (Zeijen et al., 2018).

Moreover, according to proactivity literature, when individuals identify the positive outcomes from their own behaviors and provide self-rewards for these, they are likely to experience positive affect, which will then reinforce their desired actions, energizing themselves to initiate further job crafting behaviors (Parker et al., 2010). Specifically, self-rewards represent promises people make to themselves if they persist and accomplish a particular task, spanning from quite mundane “self-gifts” such as a cup of coffee or gaming, to treating oneself to a luxury good, such as buying an expensive pair of shoes or an exclusive bottle of wine (Koch et al., 2014).

In the context of remote working, such a self-motivating process becomes particularly relevant, since goal attainment is often not clearly linked to rewards (Griffin et al., 2007; Parker et al., 2010), and individuals need to capitalize on their own self-regulation and personal resources to optimally orchestrate their job resources (Wang et al., 2020) and experience wellbeing outcomes. Hence, we expect the self-leadership strategies of self-goal setting and self-reward as mechanisms that differently empower job crafting efforts by sustaining intrinsic and extrinsic motivational processes that bolster individual proactivity. Whereas self-goal setting constitutes a behavioral strategy generating intrinsic motivational processes that may encourage action (Locke and Latham, 1990), self-reward represents an internal regulatory strategy that is supported externally (Stewart et al., 2011). This complements intrinsic motivational processes in providing the resources needed to proactively arrange the virtual work characteristics in a way that may lead to improved positive work-related outcomes.

Against this background, we propose that during remote work, employees reporting higher levels of self-goal setting and self-rewards will be more likely to initiate social interactions and proactively organize the tangible elements of their work. As such, employees who utilize these strategies are more highly motivated, which allows them to better leverage their available resources toward reorganizing their work tasks and interactions.

*Hypothesis 1*: Self-goal setting is positively associated with (a) social expansion and (b) work organization.

*Hypothesis 2*: Self-rewards are positively associated with (a) social expansion and (b) work organization.

### Job Crafting, Work Engagement, and Task Significance

Through job crafting, employees pursue positive end-states, anticipating the gain of interesting tasks and social relationships, while fulfilling their basic psychological needs in terms of autonomy and relatedness, resulting in higher work engagement (Lichtenthaler and Fischbach, 2019). Work engagement refers to a positive, fulfilling, work-related state of mind characterized by high levels of energy, dedication, and absorption in one’s work (Schaufeli et al., 2019). In the context of remote work, engaging in informal communication with colleagues has been shown to be positively related to job satisfaction (Fay and Kline, 2011), where the initiation of social interactions can reduce loneliness due to the reduction of informal social exchanges (Wang et al., 2020). Similarly, employees who are better able to organize the tangible elements of their remote work create additional resources by optimally configuring the resources they already have; hence, creating efficient work processes that positively impact their energy levels and eventually foster work engagement.

Based on these arguments and drawing on meta-analytic evidence supporting the positive link between approach-oriented job crafting and work engagement (Rudolph et al., 2017; Lichtenthaler and Fischbach, 2019; Zhang and Parker, 2019), we expect remote working job crafting to be positively linked to work engagement.

*Hypothesis 3*: (a) Social expansion and (b) work organization are positively associated with work engagement.

Overall, adopting a self-influencing perspective to the management of one’s work motivation and job characteristics (Manz, 1986), we expect that job crafting will mediate the relationship between self-leadership and work engagement. In the context of a relative absence of immediate external constraints (Thoresen and Mahoney, 1974), such as during remote working, individuals who establish targets for their work and build their own intrinsic motivational drivers will benefit from higher resource availability (Hobfoll, 2002) that can be invested to redesign one’s job to make it more organized and proactively create a social psychological work context contributing to the natural enjoyment of task performance (Manz, 1986). Hence, we propose self-leadership as a strategy that provides remote workers the inner motivation and focus to alter their environment proactively through job crafting, thereby enabling higher work engagement. In support of this, previous research in non-remote-work contexts shows that when employees use self-management strategies, they create a more resource-rich work environment, which in turn initiate a motivational process whereby employees are more engaged in their work (Xanthopoulou et al., 2009; Breevaart et al., 2014):

*Hypothesis 4*: (a) Social expansion and (b) work organization mediate the relationship between self-goal setting and work engagement.

*Hypothesis 5*: (a) Social expansion and (b) work organization mediate the relationship between self-rewards and work engagement.

During the pandemic, remote workers found themselves separated physically from their colleagues, customers, and normal workplace, alone with their computers, sporadically touching base remotely with those they used to see regularly (Gino and Cable, 2020). In a context where social gatherings have been forbidden, even limited social resources can have had strong positive effects on positive work outcomes (Wang et al., 2020), helping employees re-establish the purpose and value in their work tasks. A general tenet of job crafting research is that employees who craft their work make it more significant and meaningful, crafting more interesting job tasks, and inspiring relationships (Wrzesniewski and Dutton, 2001; Lichtenthaler and Fischbach, 2019). This happens because the meaningfulness of one’s work—that is, its purpose and value (*cf.* Grant, 2008)—acts as a lens through which employees understand and respond to their work. Through this lens, employees constantly evaluate whether they believe that their work contributes to making the world a better place, allows them to interact with people in ways that create significant contributions, or that the work provides an opportunity to earn a living (Wrzesniewski et al., 1997). As employees proactively change the task and relational components of their jobs, the emphasis of their activities and interactions shifts in ways that can profoundly impact their experience of the work and their understanding of the meaningfulness of it, which comes from employees’ perceptions of task significance (Grant, 2008; Wrzesniewski et al., 2013). Research shows that task significance can be rooted in both characteristics of the job itself (Hackman and Oldham, 1975; Grant, 2007) and relational mechanisms, with relationships being sources of task significance perceptions by connecting one’s job and actions to other people (Zalesny and Ford, 1990), while the relational aspects also enhance perceptions of social impact and social worth (Grant, 2008). Following this reasoning, employees who crafted their remote working experiences during the pandemic may have had higher chances of getting more resource value out of their set of tasks (Bruning and Campion, 2018) and build task significance as a subjective judgment that is socially constructed in interpersonal interactions (Grant, 2008). Thus, we expect employees’ job crafting activities during remote working will result in boosting task significance experienced in their work.

*Hypothesis 6*: (a) Social expansion and (b) work organization are positively associated with task significance.

Altogether, self-leadership strategies will serve to build intrinsic motivation by enhancing one’s feelings of competence and self-control (Deci, 1975; Manz, 1986) which, by enabling job crafting activities that alter job processes and the social context of work, enhance feelings of task significance. That is, based on the inner driving forces built through self-leadership, individuals will be able to alter the boundaries of their jobs in ways that allow them to experience and realize their purpose in work (Wrzesniewski et al., 2013) thereby experiencing higher task significance as a sense of purpose and beliefs in their work as an impactful activity. Hence, we further propose that job crafting mediates the role of self-leadership in enhancing task significance, with self-leadership strategies serving to create an inner driving force to craft activities that are more personally meaningful and rewarding (Manz, 1986).

*Hypothesis 7*: (a) Social expansion and (b) work organization mediate the relationship between self-goal setting and task significance.

*Hypothesis 8*: (a) Social expansion and (b) work organization mediate the relationship between self-rewards and task significance.

## Materials and Methods

### Procedure and Participants

Weekly diary data were collected over 3 weeks among employees working in a company offering services for the architecture and engineering of infrastructural networks located in Italy. At the time of the study, remote-work schedules were arranged in agreement with line managers. During the weeks of data collection, participants reported having worked remotely for, on average, 28.59 h/week (SD = 14.47).

All employees (*n* = 208) were invited to participate in the research by the HR managers, who mailed them an invitation with a link to the first online survey and information about the study. Participants were informed that their participation was voluntary and that responses would be kept confidential. Data collection started in mid-January of 2021 and lasted until mid-February of the same year. During this period, there were no significant deviations in working conditions due to the COVID-19 pandemic. Throughout the length of the study, a situation of constant high (non-critical) national alarm severely reduced travel and imposed limitations on where people could work. Survey links were sent for 3 weeks, with 1 week off between each following survey. This time frame was established with the HR function and was aimed at allowing more remote working days per employee. Along with scales to measure the study variables, the first survey also collected demographic information. Participants were asked to identify themselves using a self-generated code to match their following surveys in every survey. In each survey, participants were asked to fill in the questionnaire referring to their latest remote working experience.

The final sample consisted of 155 Italian employees (74.52% response rate), of which 53% were female (*n* = 82). Participants (*n* observations = 329) reported a mean age of 37.92 (SD = 7.33) and had worked on average 5.34 years (SD = 5.32) in the company. The majority of respondents held a masters’ degree or higher (58.1%), followed by a high school diploma (31%) or a bachelors’ degree (10.3%). A 77% of the participants had a permanent full-time contract, and 30% reported having care duties at home (referred to as “non-formal domestic work carried out for non-self-sufficient people, such as children, the elderly and the disabled”).

### Measures

All measures were administered in Italian. Scales not available in Italian were translated using the forward-backward translation method (Behling and Law, 2000). The time frame of the scales and the number of items were adapted to be answered on a weekly basis (Ohly et al., 2010). In all surveys, we asked participants to reflect upon their experiences during the past week and indicate how each item was representative of their most recent remote-work experience.

#### Weekly Self-Leadership

Weekly self-leadership during remote working was measured with five items measuring the behavioral strategies of self-goal setting (3 items, i.e., “*This week, when working remotely, I consciously had goals in mind for my work efforts*”) and self-rewards (2 items, i.e., “*This week, when working remotely, when I did something well, I treated myself to some thing or activity I especially enjoy*”) developed by Houghton and Neck (2002). Items were rated on a seven-point scale (1 = *never*; 7 = *very often*).

#### Weekly Job Crafting

Weekly job crafting during remote working was measured with nine items from the scale developed by Bruning and Campion (2018), measuring two dimensions of job crafting, namely, social expansion (3 items, i.e., “*This week, when working remotely, I actively initiated positive interactions with others at work*”) and work organization (3 items, i.e., “*This week, when working remotely, I created a structure in my work processes*”). Items were rated on a seven-point scale (1 = *never*; 7 = *very often*).

#### Weekly Work Engagement

Weekly work engagement was measured with three items from the ultra-short measure for work engagement developed by Schaufeli et al. (2019), i.e., “*This week, when working remotely*, *I felt bursting with energy*” (vigor); “*This week, when working remotely, I felt enthusiastic about my job*” (dedication); and “*This week, during remote working, I was immersed in my work*” (absorption). Participants answered on a seven-point scale (0 = *Not at all*; 6 = *To a very large degree*).

#### Weekly Task Significance

Weekly task significance was measured with three items (i.e., “*This week, when working remotely, I felt like the results of my work significantly affected the lives and well-being of other people*” from the revised Job Diagnostic Survey; Idaszak and Drasgow, 1987). Participants indicated how accurately or inaccurately each statement described their job on a seven-point scale (1 = *Very inaccurate*; 7 = *Very accurate*).

### Statistical Approach

Our data have a multilevel structure, with week-level measures (Level 1) nested within employees (Level 2). We calculated the intra-class correlations (ICC1; Bliese, 2000) for our variables before hypothesis testing. The between-persons variance for our variables varied from 74% for work organization to 52% for self-rewards, warranting an examination of our hypotheses that accounts for the variation between clusters in our variables.

We conducted multilevel confirmatory factor analysis (MCFA) in *Mplus* version 8.4 (Muthén and Muthén, 2012) to examine the factorial validity of our measures and estimate their multilevel composite reliabilities (*ω*; Geldhof et al., 2014). A six-factor model was specified at both the within- and between levels, estimating the loadings of respective items on the latent variables (i.e., self-goal setting, self-rewards, social expansion, work organization, work engagement, and task significance). Multilevel composite reliability (ω) was estimated at both levels of analysis using estimated level-specific factor loadings and residual variances. Correlations among the latent factors at both levels were freely estimated.

To test our hypotheses, we used multilevel modeling in *Mplus* version 8.4 (Muthén and Muthén, 2012). Multilevel modeling is based on decomposing the data into within-person (week-level) and between-person (person-level) parts and modeling each of these parts with their own model (Muthén and Asparouhov, 2008). Following previous research, we used observed variables to avoid overly complex modeling (*cf.* Bakker and Oerlemans, 2019; Chong et al., 2020; Sonnentag et al., 2021).

In our analysis, we controlled for age, which has been shown to relate to job crafting (Kooij et al., 2017b). Gender was also controlled for since it is likely that the pandemic differently affected men and women (Kniffin et al., 2021). Additionally, the number of remote working hours in the previous week was controlled for, since this may have affected the extent to which employees felt the need to engage in self-leadership and job crafting during remote working. Age and gender were specified as between-level variables since they only had between variance and were centered at the grand mean to aid interpretation (Preacher et al., 2010). Weekly self-leadership (self-goal setting and self-rewards), job crafting (social expansion and work organization), work engagement, and task significance, as well as number of remote working hours in the previous week, were not specified as either within or between variables and were modeled at both levels as their variance was partitioned into within- and between components (Preacher et al., 2010). This procedure implies that the weekly level variables are implicitly centered at the person-level (Preacher et al., 2010), removing the between-person variance from the within-person part of the model (Sonnentag et al., 2021).

## Results

### Preliminary Analyses and Descriptive Statistics

Results from the MCFA showed that the six-factor model estimated simultaneously at both levels fit the data well, *χ*^2^_(208)_ = 317.70, *p* < 0.001; root-mean-square error of approximation (RMSEA) = 0.04, and standardized root-mean-square residual (SRMR) SRMR _within_ = 0.06; SRMR _between_ = 0.07, where RMSEA and SRMR values of 0.08 or less indicate adequate fit (Hu and Bentler, 1999). All indicators significantly loaded on their respective factors. An alternative model in which the items from the two self-leadership strategies (self-goal setting and self-rewards), the two job crafting dimensions (social expansion and work organization), and the two outcome variables loaded into a three-factor (*χ*^2^_(232)_ = 859.79, *p* < 0.001; RMSEA = 0.09, SRMR _within_ = 0.47; SRMR _between_ = 0.58) had a poorer fit to the data, supporting six factors as distinct.

Table 1 shows descriptive statistics, within- and between-persons reliabilities, intra-class correlations coefficients (ICCs) for weekly measures, and correlations among the variables. The within-person reliabilities were acceptable, showing the ability of the scales to detect changes for a person over weeks. Similarly, between-person reliabilities were acceptable and able to discriminate different people’s weekly average measures.

**Table 1:** Descriptive statistics and correlations among the study variables.

Variable	*M* (SD)_B_	*ω*_B_	*M* (SD)_W_	*ω*_W_	ICC	1	2	3	4	5	6	7	8	9
Age	36.96 (11.16)	–	–	–	–	(−)	0.14^*^	0.09	0.19^**^	0.04	0.09	0.11	0.17^**^	0.04
Gender	1.47 (0.50)	–	–	–	–	0.14^*^	(−)	−0.14^**^	−0.21^**^	0.03	−0.13^*^	−0.22^**^	−0.10	0.14^*^
Remote working hours	28.59 (13.63)	–	28.59 (14.47)	–	–	0.09	−0.15^**^	(−)	0.19^**^	0.08	0.24^**^	0.29^**^	0.21^**^	0.01
Self-goal setting	5.94 (0.97)	0.98	5.94 (1.07)	0.68	0.66	0.20^**^	−0.23^**^	0.23^**^	(−)	0.17^**^	0.49^**^	0.72^**^	0.48^**^	0.23^**^
Self-rewards	3.47 (1.48)	0.97	3.47 (1.71)	0.83	0.52	0.04	0.03	0.10	0.21^**^	(−)	0.29^**^	0.19^**^	0.26^**^	0.09
Social expansion	5.28 (1.28)	0.98	5.28 (1.38)	0.74	0.74	0.10	−0.14^*^	0.27^**^	0.53^**^	0.32^**^	(−)	0.49^**^	0.48^**^	0.32^**^
Work organization	5.67 (1.11)	0.96	5.67 (1.19)	0.68	0.74	0.11^*^	−0.23^**^	0.32^**^	0.79^**^	0.22^**^	0.51^**^	(−)	0.45^**^	0.21^**^
Work engagement	4.90 (1.33)	0.97	4.90 (1.43)	0.78	0.72	0.19^**^	−0.11^*^	0.24^**^	0.54^**^	0.28^**^	0.51^**^	0.48^**^	(−)	0.14^*^
Task significance	5.21(1.12)	0.91	5.21 (1.25)	0.58	0.63	0.04	0.15^**^	0.03	0.27^**^	0.14^*^	0.36^**^	0.23^**^	0.15^**^	(−)

*Between-person (B) correlations below the diagonal (n = 155). Within-person (W) correlations above the diagonal (n = 329). ω = Reliability of change; Remote working hours = Number of remote working hours during the previous week*.

*ICC = intra-class correlation coefficient. Gender: 1 = Female; 2 = Male*.

* *p < 0.05*;

** *p < 0.01.*

### Hypotheses Testing

**Figure 2:**
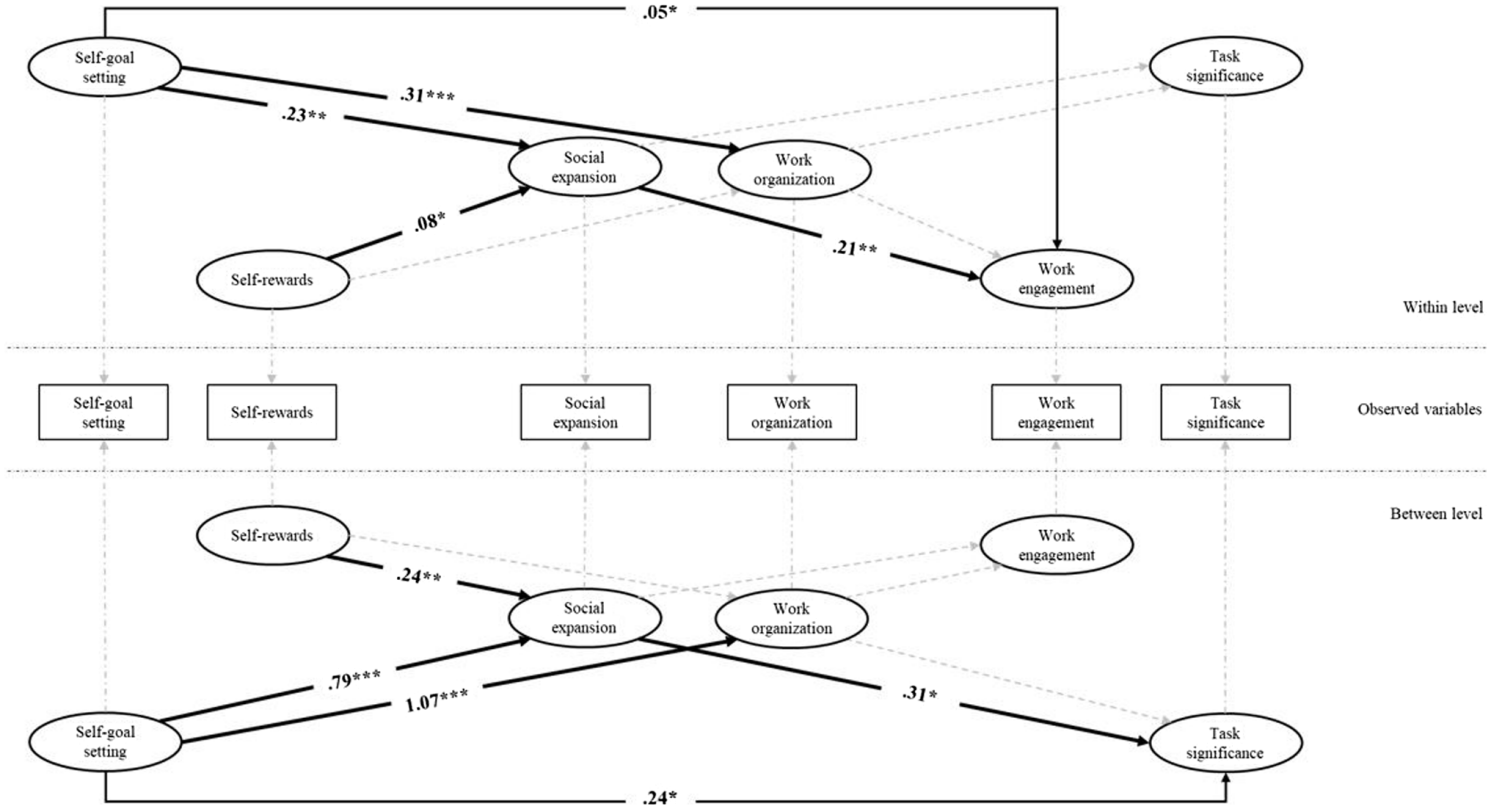
Result of the multilevel model. Bold arrows represent significant paths. Non-standardized significant estimates are displayed. The results account for the role of number weekly remote working hours. Control variables (age and gender), related paths, and estimated paths from independent variables to outcome variables are not displayed for the sake of clarity. Only indirect paths that are significant are displayed. ^***^p ≤ 0.001, ^**^p ≤ 0.01, and ^*^p ≤ 0.05.

We tested our hypotheses in a model with similar paths at the within- and between-person levels, except for the paths involving gender and age modeled only at the between level. Control variables included gender, age, and number of remote working hours in the previous week. The multilevel model fit well to the data: *χ*^2^_(24)_ = 53.42, *p* < 0.001; RMSEA = 0.06, SRMR _within_ = 0.07; SRMR _between_ = 0.07. Figure 2 shows the unstandardized estimates and significance levels of the significant relationships found.

#### Self-Leadership ➔ Job Crafting

Hypothesis 1 proposed that self-goal setting is positively associated with job crafting in terms of (a) social expansion and (b) work organization. As shown in Table 2, on weeks when employees reported higher self-goal setting, they engaged more often in social expansion (*estimate* = 0.23, *se* = 0.09, *t* = 2.62, *p* ≤ 0.01) and proactively organized their work processes more often (*estimate* = 0.31, *se* = 0.06, *t* = 5.19, *p* < 0.001) while working remotely. The same relationships were also significant when examining differences between employees, with self-goal setting being significantly positively associated with both social expansion (*estimate* = 0.79, *se* = 0.12, *t* = 6.72, *p* < 0.001) and work organization (*estimate* = 1.07, *se* = 0.09, *t* = 11.59, *p* < 0.001). Hence, Hypothesis 1 is confirmed.

**Table 2:** Unstandardized coefficients from multilevel path modeling predicting social expansion and work organization.

	Social expansion				Work organization			
Variable	Est.	SE	*t*	*p*	Est.	SE	*t*	*p*
*Between-person level*								
Intercept	−0.23	0.66	−0.34	0.73	−0.92	0.56	−1.65	0.10
Self-goal setting	0.79	0.12	6.72	<0.001	1.07	0.09	11.59	<0.001
Self-rewards	0.24	0.09	2.81	0.01	0.06	0.07	0.89	0.38
Residual variance	0.81	0.20	4.04	<0.001	0.21	0.07	3.05	0.002
*Within-person level*								
Self-goal setting	0.23	0.09	2.62	0.001	0.31	0.06	5.19	<0.001
Self-rewards	0.08	0.04	1.93	0.05	0.04	0.04	1.06	0.29
Residual variance	0.45	0.07	6.38	<0.001	0.33	0.05	7.14	<0.001

*Estimates are unstandardized, resulting from one overall analysis including the prediction of the different self-leadership strategies on work engagement and task significance via job crafting behaviors*.

Hypothesis 2 proposed that self-rewards are positively associated with job crafting in terms of (a) social expansion and (b) work organization. At the within level, results showed that on weeks when employees reported higher self-rewards, they engaged more often in social expansion (*estimate* = 0.08, *se* = 0.04, *t* = 1.93, *p* ≤ 0.05) but did not proactively organize their work processes more often while working remotely. When examining differences between employees, self-rewards resulted significantly positively associated with social expansion (*estimate* = 0.24, *se* = 0.09, *t* = 2.81, *p* = 0.005) but not with work organization. Hence, Hypothesis 2a is confirmed while Hypothesis 2b is rejected.

#### Job Crafting ➔ Work Engagement

Hypothesis 3 stated that job crafting behaviors in terms of (a) social expansion and (b) work organization are positively associated with work engagement. At the within level, results (see Table 3) showed that on weeks when employees reported higher social expansion behaviors during remote working, they experienced higher work engagement (*estimate* = 0.21, *se* = 0.08, *t* = 2.53, *p* = 0.01), but no significant relationships were found with work organization. At the between level, neither social expansion nor work organization were significantly associated with work engagement. Accordingly, Hypothesis 3a is confirmed only at the within level, while Hypothesis 3b is rejected.

**Table 3:** Unstandardized coefficients from multilevel path modeling predicting work engagement and task significance.

	Work engagement				Task significance			
Variable	Est.	SE	*t*	*p*	Est.	SE	*t*	*p*
*Between-person level*								
Intercept	−0.55	0.88	−0.62	0.53	2.98	0.87	3.43	0.001
Self-goal setting	0.78	0.44	1.78	0.08	0.14	0.45	0.31	0.76
Self-rewards	0.13	0.10	1.39	0.16	0.05	0.10	0.50	0.62
Social expansion	0.21	0.15	1.39	0.17	0.31	0.12	2.53	0.01
Work organization	−0.12	0.34	−0.37	0.72	−0.07	0.35	−0.20	0.84
Residual variance	0.83	0.13	6.60	<0.001	0.80	0.11	6.99	<0.001
*Within-person level*								
Self-goal setting	0.16	0.11	1.45	0.15	0.07	0.10	0.66	0.51
Self-rewards	0.06	0.05	1.29	0.20	−0.07	0.05	−1.25	0.21
Social expansion	0.21	0.08	2.56	0.01	0.13	0.10	1.31	0.19
Work organization	0.13	0.09	1.53	0.13	0.08	0.10	0.77	0.44
Residual variance	0.47	0.08	5.66	<0.001	0.55	0.07	7.48	<0.001

*Estimates are unstandardized, resulting from one overall analysis including the prediction of the different self-leadership strategies on work engagement and task significance via job crafting behaviors*.

#### Self-Leadership ➔ Job Crafting ➔ Work Engagement

The indirect effects of self-goal setting (Hypothesis 4) and self-rewards (Hypothesis 5) on work engagement *via* remote working job crafting (a—social expansion; b—work organization) were tested with M*plus* following the procedure by Preacher et al. (2010) and the Monte Carlo method with 20,000 repetitions (Preacher and Selig, 2010). As reported in Table 4, weekly social expansion significantly mediated the effect of self-goal setting on work engagement (*estimate* = 0.05, *se* = 0.02, *t* = 2.07, *p* = 0.04; 95%CI [0.01, 0.11]), while all the other indirect effects were not significant. Hence, Hypothesis 4a is confirmed only at the within level, while Hypothesis 4b, and Hypotheses 5a and 5b are rejected.

**Table 4:** Indirect effects of self-leadership on work engagement *via* job crafting.

	Between level				Within level			
Indirect effect x ➔ m ➔ y	Est.	SE	*t*	*p*	Est.	SE	*t*	*p*
Self-goal setting ➔ social expansion ➔ work engagement	0.17	0.12	1.40	0.16	0.05	0.02	2.07	0.04
Self-goal setting ➔ work organization ➔ work engagement	−0.16	0.33	−0.49	0.62	0.04	0.03	1.45	0.15
Self-rewards ➔ social expansion ➔ work engagement	0.05	0.04	1.23	0.22	0.02	0.01	1.45	0.15
Self-rewards ➔ work organization ➔ work engagement	−0.01	0.02	−0.44	0.66	0.01	0.01	1.00	0.32

*Estimates are unstandardized, resulting from one overall analysis including the prediction of the different self-leadership strategies on work engagement and task significance via job crafting behaviors*.

#### Job Crafting ➔ Task Significance

Hypothesis 6 proposed that job crafting behaviors in terms of (a) social expansion and (b) work organization are positively associated with task significance. As it can be seen in Table 3, at the within level no significant relationships were found. Differently, when considering differences between employees, results showed that those reporting higher social expansion while working remotely also scored higher in task significance during the weeks (*estimate* = 0.31, *se* = 0.13, *t* = 2.44, *p* = 0.02), while no significant relationships were found for work organization. Hence, Hypothesis 6a is confirmed only at the between level while Hypothesis 6b is rejected.

#### Self-Leadership ➔ Job Crafting ➔ Task Significance

As displayed in Table 5, the test of the indirect effect of self-leadership on task significance *via* job crafting (Hypothesis 7 and 8) showed that, only at the between level, self-goal setting was significantly indirectly linked to higher task significance *via* social expansion behaviors (*estimate* = 0.24, *se* = 0.10, *t* = 2.42, *p* = 0.02; 95%CI [0.02, 0.50]). All the other indirect effects were not significant. Accordingly, Hypothesis 7a is supported at the between level, while Hypothesis 7b, 8a, and 8b are rejected.

**Table 5:** Indirect effects of self-leadership on task significance *via* job crafting.

	Between level				Within level			
Indirect effect x ➔ m ➔ y	Est.	SE	*t*	*p*	Est.	SE	*t*	*p*
Self-goal setting ➔ social expansion ➔ task significance	0.24	0.10	2.42	0.02	0.03	0.03	1.18	0.24
Self-goal setting ➔ work organization ➔ task significance	−0.14	0.36	−0.39	0.69	0.03	0.03	0.83	0.41
Self-rewards ➔ social expansion ➔ task significance	0.07	0.04	1.85	0.06	0.01	0.01	1.08	0.28
Self-rewards ➔ work organization ➔ task significance	−0.01	0.01	−0.36	0.72	0.01	0.01	0.67	0.50

*Estimates are unstandardized, resulting from one overall analysis including the prediction of the different self-leadership strategies on work engagement and task significance via job crafting behaviors*.

## Discussion

### Theoretical Contributions

Results of the current research advance proactivity literature by showing that self-leadership enables the proactive initiation of social interactions during remote working and that some proactive strategies are more effective in driving certain downstream outcomes than others. Results provide further support for the theoretical link between job crafting and work engagement (Zhang and Parker, 2019) and (to the best of our knowledge) provide the first support for the effect of job crafting on task significance during remote working. The study also contributes to job crafting literature by providing evidence for the role of a specific form of job crafting (i.e., social expansion) as a mediating mechanism between self-leadership and critical work outcomes. Additionally, results show that the proactive and self-regulatory processes occurring during remote work are not consistent across different level of analysis, suggesting that these processes unfold differently when considering differences in self-regulatory efforts between individuals or changes in these efforts over time within a same person.

Namely, social expansion mediated the relationship between self-goal setting and task significance at the between level, and the relationship between self-goal setting and work engagement at the within level, but no other indirect effects were supported. These results enrich self-leadership and job crafting literature by showing that self-goal setting is an effective driver of work engagement and task significance through social expansion and that self-rewards and work organization may be less effective in driving the critical work outcomes explored in this study. Additionally, given that the indirect effect of self-goal setting on work engagement through social expansion was only significant at the within level, this implies that work engagement may be more fluid within person during the time-period explored in our study, and conversely, that task significance may require longer-term exploration and be less fluid in the short-term. This notion is further supported in that no variables predicted task significance at the within level, and no variables predicted work engagement at the between level.

While self-rewards predicted social expansion at both levels, no other significant relationships were found between this predictor and any other variable at either level. From a theoretical perspective, these findings emphasize that while self-rewards represent a self-influence strategy, such a strategy then triggers relational mechanisms through which remote workers can experience their actions as related and connected to other people (Ryan and Deci, 2000). Hence, workers rewarding themselves for their own good work have a higher focus on how their work results may fit in with overall work goals. Then, when they feel such goals have been attained, they look for ways to consolidate and link their individual contribution to others at work. However, results also suggest that this type of self-leadership may not be an effective means of driving downstream work outcomes, perhaps because the rewards employees provide for themselves may not always be proximally related to the workplace. For example, while people may reward themselves for accomplishing a work task and reach out to a colleague to share such an achievement, the motivational driver coming from the experience of rewarding oneself may be experienced as personal, rather than professional, and may not be leveraged as a mechanism for driving downstream significance in their work or further engagement in other work-related tasks.

Meanwhile, self-goal setting had significant relationships with both social expansion and work organization at the within- and between levels, as well as the indirect relationships already discussed. These findings provide further support for the robustness of goal-setting theory (Locke and Latham, 1990) for driving positive work outcomes such as work engagement (Weintraub et al., 2021). These results support the notion that self-goal setting can help sustain more fluid variables such as work engagement by providing the self-motivation and self-direction needed to facilitate behaviors that may be necessary yet undesirable to accomplish work tasks (Bakker and van Woerkom, 2017). Furthermore, results suggest that self-goal setting can help drive downstream effects which are more stable and may take longer to develop such as task significance. In the context of being isolated at home during a global pandemic while working, these results suggest that self-goal setting allowed workers to stay connected to their co-workers through social expansion, which may have led to the fulfillment of the need for relatedness and a feeling that the work their community does is more meaningful.

Finally, while work organization was predicted by self-goal setting at the within-level and at the between level, it was not a predictor of either work outcome explored in this study. From a theoretical standpoint, it may be that the way one organizes their work does not affect the way the work itself is perceived or inspires workers to engage more fervently with their work tasks. Instead, it may be more of a logistical strategy than one which drives changes in states such as engagement or attitudes about one’s work such as task significance.

### Practical Implications

From a practical standpoint, the current study had clear takeaways which can be leveraged by individuals, teams, and organizations. First, if the aim is to drive improvements in task significance and work engagement in a remote-work context, organizations should encourage employees to set goals for themselves and that at least a portion of these goals should be related to social expansion. For example, employees may be encouraged to meet with colleagues to discuss work tasks and how they might collaborate on projects. This encouragement could be communicated verbally by leadership or utilize mechanisms like nudges (Thaler and Sunstein, 2008) in which goal-setting frameworks are introduced and encouraged *via* e-mail or app-delivered reminders (Weintraub et al., 2021). Such strategies can teach individuals the skills needed to set goals for themselves while also preserving autonomy and the self-leadership aspect of this strategy.

Meanwhile, our results suggest that self-rewards may be an effective strategy for driving social expansion but are not a potent enough intervention to influence the work outcomes of task significance or work engagement. Therefore, if companies have limited resources and need to choose between encouraging self-rewards or self-goal setting, self-goal setting has the potential to have more incremental value. However, if organizations are struggling with building a sense of community, self-rewards may still be an effective means of driving social expansion within organizations. Similarly, our findings suggest that work organization may not be worth spending organizational resources on in situations where building work engagement or task significance are the goals.

### Limitations and Future Research

While this study does provide many theoretical and practical contributions, like all studies, there are limitations that should also inspire future research. First, it must be noted that this study was conducted during a global pandemic. As such, there may be a distinction between working remotely in this context compared to remote work in the future. For example, our findings regarding work organization may have limited generalizability in that workers may have less autonomy over managing behavior or physical surroundings to increase structural job resources since they are unlikely to have planned to work in the conditions which were present in the current study. For example, workers in this study could have typically worked in traditional in-person office settings but had to quickly shift to working from home due to COVID-19. As such, rather than choosing living situations which they could have more control over physical surroundings, they were likely forced into spaces where they had not planned to do work, and which may not be conducive to working (i.e., sharing small spaces where roommates are also working or where children are home from school). Future research should replicate the current study and ask individuals about their *typical* work environment, more detailed accounts of their work-from-home setup (e.g., whether they have their own private home-office or work in the same room with others), and whether they feel they have the resources to accomplish their work properly while working remotely.

It should be noted that our sample size was relatively small and that all participants came from a single company, which may limit the generalizability of our findings. Additionally, mean values for self-rewards in our sample were relatively low (i.e., mean = 3.47, range = 1–7), especially when compared with other variables (i.e., self-goal setting mean = 5.95). This implies that self-rewards were not very commonly used in this sample, which could have impacted our ability to find support for our hypotheses. Conversely, participants in our sample frequently reported generally high self-goal setting and job crafting behaviors. While the analyses we adopted focused on how deviations from individual means are associated with high/lower outcomes, these aspects should be considered when evaluating our findings. As such, future research should intentionally recruit a larger number of participants, from multiple organizations, with a wider range of self-rewarding and proactive behaviors, to better examine the relationships of interest.

This study focused on self-leadership as an antecedent of job crafting behaviors aiding positive remote working experiences. There are, however, other personal attributes and contextual factors that may influence self-regulation strategies and processes that we did not include (e.g., trait emotional stability, conscientiousness, work-related self-efficacy, level of work autonomy, presence of clearly specified goals, and fit-discrepancy between self-settled and organizational goals). Similarly, in this research, we focused on psychological work-related outcomes and did not investigate any potential effect on employees’ health. Future research could examine the role of personal attributes and contextual variables in explaining proactivity and its effects during remote working. The effect of self-leadership strategies and job crafting on other work outcomes such as objective performance and wellbeing, including health indicators, should also be explored.

In the current study, we did not examine the types of rewards or goals that workers set or provided for themselves. For example, with regard to rewards, some workers may have been providing big, expensive rewards for themselves while others may have rewarded themselves with smaller things, or even with different categories of rewards (i.e., monetary rewards vs. allowing themselves to eat a treat they enjoy vs. giving themselves time to relax). Likewise, the content of goals has been shown in previous research to have differential effects on downstream variables such as work engagement (Weintraub et al., 2021). Therefore, future research should aim to utilize a mixed-methods approach in which quantitative and qualitative aspects of goals and rewards can be further examined.

Finally, all the variables in our study were self-assessed, and the design of this study was observational in nature, which may lead to the risk of common method bias (Podsakoff et al., 2003). However, it may be argued that employees themselves are best suited to self-report their self-leadership processes because they are the ones who are aware of how they proactively manage their motivational processes while working remotely. Moreover, evidence from research shows high agreement between self- and peer-ratings of approach-oriented job crafting (Tims et al., 2012). Also, multilevel confirmatory factor analyses revealed a good fit, indicating construct validity, which represents one way to rule out substantial method effects (Conway and Lance, 2010). Still, the fact that our variables were assessed at the same time point in time, at the end of the week, makes it important for future studies to employ experimental designs in which interventions can be further assessed for causality and across a longer period of time to examine the longevity of the potency of their effects. Given the potential for the fluctuation in variables such as work engagement within day, future research could also record more frequent measurements to further examine how these strategies might affect job crafting behaviors within the same day (daily diary studies) as well as over longer periods of time, which could also differentially affect work outcomes.

## Conclusion

This study explored the effects of self-leadership practices on key work outcomes in a remote-work environment during a global pandemic and the mediating role of job crafting on this relationship. In particular, the study explored social expansion and work organization mediating the relationships between self-goal setting and self-rewards predicting work engagement over time. It was also the first known study to explore the effect of job crafting on task significance during remote working. Overall, our results provided support for these theoretical assertions. Although nuanced, findings suggest that self-goal setting is a particularly potential self-leadership strategy that leads to job crafting and the work outcomes of task significance and work engagement. These results also provide practical implications for self-goal setting as a self-leadership strategy that should be encouraged by organizations. Future research should employ mixed-method experimental designs which test interventions and examine how individual differences may affect these relationships over time.

## Data Availability Statement

The raw data supporting the conclusions of this article will be made available by the authors, without undue reservation.

## Ethics Statement

Ethical review and approval were not required for the study on human participants in accordance with the local legislation and institutional requirements. The patients/participants provided their written informed consent to participate in this study.

## Author Contributions

AC and JW contributed to conception and design of the study and wrote the first draft of the manuscript. AC organized the database and performed the statistical analysis. All authors contributed to the article and approved the submitted version.

## Conflict of Interest

JW was employed by the Flow Group, LLC.

The remaining author declares that the research was conducted in the absence of any commercial or financial relationships that could be construed as a potential conflict of interest.

## Publisher’s Note

All claims expressed in this article are solely those of the authors and do not necessarily represent those of their affiliated organizations, or those of the publisher, the editors and the reviewers. Any product that may be evaluated in this article, or claim that may be made by its manufacturer, is not guaranteed or endorsed by the publisher.
